# Assessment of Joystick control during the performance of powered wheelchair driving tasks

**DOI:** 10.1186/1743-0003-8-31

**Published:** 2011-05-24

**Authors:** Gianluca U Sorrento, Philippe S Archambault, François Routhier, Danielle Dessureault, Patrick Boissy

**Affiliations:** 1 School of Physical & Occupational Therapy, McGill University, Montréal, Canada; 2 Centre for Interdisciplinary Research in Rehabilitation of Greater Montreal (CRIR), Jewish Rehabilitation Hospital, Montréal, Canada; 3 Center for Interdisciplinary Research in Rehabilitation and Social Integration (CIRRIS), Institut de réadaptation en déficience physique de Québec, Québec, Canada; 4 Technical Aids Department, Centre de réadptation Lucie-Bruneau, Montréal, Canada; 5 Research Centre on Aging, CSS-IUGS, Sherbrooke, Canada; 6 Faculty of Medecine and Health Sciences Department of Surgery, Université de Sherbrooke, Sherbrooke, Canada

## Abstract

**Background:**

Powered wheelchairs are essential for many individuals who have mobility impairments. Nevertheless, if operated improperly, the powered wheelchair poses dangers to both the user and to those in its vicinity. Thus, operating a powered wheelchair with some degree of proficiency is important for safety, and measuring driving skills becomes an important issue to address. The objective of this study was to explore the discriminate validity of outcome measures of driving skills based on joystick control strategies and performance recorded using a data logging system.

**Methods:**

We compared joystick control strategies and performance during standardized driving tasks between a group of 10 expert and 13 novice powered wheelchair users. Driving tasks were drawn from the Wheelchair Skills Test (v. 4.1). Data from the joystick controller were collected on a data logging system. Joystick control strategies and performance outcome measures included the mean number of joystick movements, time required to complete tasks, as well as variability of joystick direction.

**Results:**

In simpler tasks, the expert group's driving skills were comparable to those of the novice group. Yet, in more difficult and spatially confined tasks, the expert group required fewer joystick movements for task completion. In some cases, experts also completed tasks in approximately half the time with respect to the novice group.

**Conclusions:**

The analysis of joystick control made it possible to discriminate between novice and expert powered wheelchair users in a variety of driving tasks. These results imply that in spatially confined areas, a greater powered wheelchair driving skill level is required to complete tasks efficiently. Based on these findings, it would appear that the use of joystick signal analysis constitutes an objective tool for the measurement of powered wheelchair driving skills. This tool may be useful for the clinical assessment and training of powered wheelchair skills.

## Background

Impaired mobility, secondary to health conditions such as spinal cord injury, stroke, rheumatoid arthritis, amputation and complication from diabetes, to name a few, are often accompanied by environmental barriers which can restrict activities of daily living [1] and impact the individual's quality of life [2-5]. In this context, the use of a powered wheelchair (PW) by those who face such challenges can be highly beneficial [5-8]. The benefits of PW mobility span a large spectrum of the demographic across age groups and health conditions [9-12]. It can also provide psychological benefits, as users generally report feeling a greater sense of independence [13]. Yet, despite the advantages of using a PW, its maneuverability and speed can pose challenges to the user [14], particularly when negotiating uneven surfaces encountered daily, such as road potholes and sidewalks [15,16]. Therefore, it is essential that PW users develop the skill-set necessary to operate the wheelchair safely and competently. It is equally important to evaluate and monitor the user's progress of driving skills [11]. In recent years, assessments such as the Wheelchair Skills Test (WST-P) [17,18] have provided valid criteria for the competent and safe execution of PW driving related tasks [18,19]. These assessments have shown to be sensitive to change, valid, and reliable as improvements in the efficacy and safety of both manual and powered wheelchair operators were observed after a wheelchair skills training program [17,20-22]. However, this evaluation process is mainly based on the clinical observations of a trained evaluator.

Implementing PWs with sensors and collecting data during standardized driving tasks could provide objective and sensitive measures for the control and the movement of the PW, thereby complementing observation-based findings [8,23,24]. Specifically, they could serve as insightful outcome measures of how well users maneuver the wheelchair to complete a wide array of tasks across varying levels of difficulty [24-26].

In this study, we adopted this approach to evaluate the PW driving skills of novice and expert PW users. The primary objective of this study was to explore the discriminate validity of outcome measures of driving skills based on joystick control strategies and performance recorded using a data logging system.

## Methods

### Participants

An experimental group consisted of 10 individuals who require daily use of a powered wheelchair (PW), and had more than six months of PW driving experience at the time of testing. Participants in this group had varying degrees of physical impairment and various diagnoses (See Table 1). A group of 13 individuals free of impairment were recruited as novice participants. These novice participants were recruited based on having no experience operating a PW. All expert wheelchair users provided and operated their own rear-wheeled Oasis II (Orthofab, Canada) PW model. Novice users were given a PW of the same model to operate in the study. All participants used a standard hand-controlled joystick that was modified for data collection. The investigators made arrangements to ensure seating posture and joystick positions for each participant were as comfortable as possible. All subjects were right handed, yet the joystick could be mounted on the left or right to accommodate the handedness of participant. The ethics review boards of the Institut de réadaptation en déficience physique du Québec (IRDPQ) and the Center for interdisciplinary research in rehabilitation of the greater Montreal (CRIR) approved the study and all participants provided their informed consent.

**Table 1:** Demographic summary of expert and novice groups

	Gender	Age (years) Mean (±SD)	Diagnosis	PW Wheelchair Experience (years) Mean (±SD)
*Experts *(n = 10)				
E1	F	58	Neuropathy	17
E2	F	57	Neuropathy	15
E3	M	57	Neuropathy	3
E4	M	34	Neuropathy	5
E5	F	43	Neuropathy	1
E6	M	26	Neuropathy	3
E7	F	57	Neuropathy	10
E8	M	71	Diabetes (Type II)	1
E9	M	62	Neuropathy	7
E10	M	63	Spinal cord injury	6
	6M/4F	52.8 (14.0)		6.8 (5.6)
*Novice *(n = 13)				
	5M/8F	24.4 (5.4)		

Novice participants were free of any neurological impairment and had no prior PW driving experience.

### Tasks and Evaluation

Participation from both the expert and novice groups consisted of executing tasks drawn from the Wheelchair Skills Test (WST, PW version 4.1) [17]. In its entirety, the WST-P is a list of 32 tasks (named "skills" by the WST authors) that evaluates the user's general capacity to use a PW, paying close attention to their driving skills performance and safety practices. The first section of the WST-P is intended to test the participant's capacity to operate basic functions of the wheelchair and controls (e.g. operating tilt and recline, charging batteries, operating the joystick). For example, participants are asked to turn the wheelchair on and off, select different speeds (drive modes), and recharge the PW's power source. The rest of the evaluation consists of driving tasks including reversing, turning, and negotiating maneuvers in tight quarters. Each participant's mobility is assessed within and about the wheelchair through transferring, changing posture, and reaching for objects. Central to this study is assessing how well participants operate the PW joystick. To investigate this, we selected six of the WST-P tasks for data collection and analysis. These tasks were selected since they required driving the PW with at least a minimal amount of maneuvering, such as turning or backward driving. The selected tasks were:

#### Rolls Backward 5 m

Participants are evaluated based on how well they operate the PW in the reverse direction while maintaining a straight trajectory and traveling at an appropriate speed. Participants were asked to place their PW in front of a pre-marked starting line and were instructed to move the PW backward until they reached a finishing marker placed on the floor 5 meters directly behind them.

#### Turns 90° While Moving (forward and backward; right and left)

**Figure 1:**
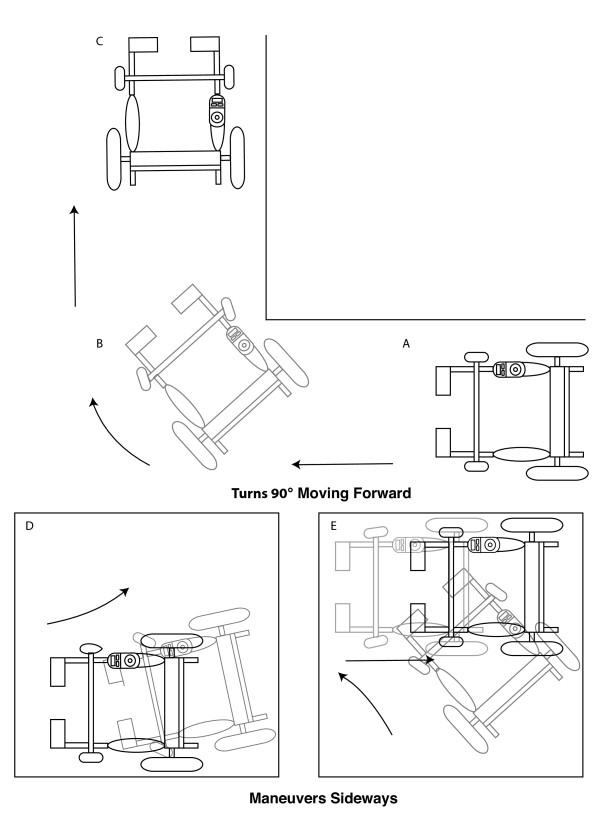
***Turns 90° While Moving Forward *and *Maneuvers Sideways *tasks**. A: For the *Turns 90° While Moving Forward *task, participants initially started with the wheels ahead of a pre-marked start position, B: they executed a 90° turn C: and continued to end marker. D: Typical starting point for a participant in the *Maneuvers Sideways *task. The PW is initially stationed on one side of the testing area. Participants then attempt to maneuver the PW using reverse, forward, and lateral movements until they are able to move to the opposite side of the testing area (E).

This task evaluated the user's ability to turn the PW left or right, while traveling in the forward or backward direction. Participants placed the PW's rear wheels in front of a starting marker on the ground. They were instructed to proceed forward and then turn right at the corner, thereby executing a 90° turn to continue until finally reaching the finishing marker. The total travel distance was approximately 6 meters (see Figure 1A-C).

#### Turns 180° in Place (right and left)

This task was employed for assessing how well the user could change directions in a spatially confined area. The participants placed themselves in the middle of a pre-marked 1.5 m^2 ^area. They were then instructed to rotate the chair 180°, trying to keep all parts of the wheelchair within the pre-marked square. Due to the PW's size and rear-traction, it does not pivot around its center. Therefore, success in this task requires skillful execution of rotary movements in forward and backward directions.

#### Maneuvers Sideways (right and left)

This task examined how well the user could place the PW from one side of a confined area (i.e. against a wall) to within 10 cm of the opposite side, as to simulate approaching and positioning the PW near a bed or chair for transferring. Participants began with one side of the PW placed adjacent to a wall. They then executed a series of maneuvers in an attempt to place the opposite side of the PW to the opposite wall (see Figure 1D-E). As in the 180° turning task, subjects were instructed to avoid crossing the testing boundaries.

#### Gets Through Hinged Door in Both Directions

This task was used to assess how well PW users could negotiate from one room to another by opening a door, entering the adjacent room and closing the door behind them. This task had two variations; the first involved initially pushing the door open, moving through the doorway and pushing the door closed on the other side. The second variation involved pulling the door open towards the chair, proceeding through the doorway, and finally reaching for the doorknob to pull the door closed.

Prior to the execution of each task, participants were given clear instructions regarding what was expected for successful task completion, outlining the boundaries that the participants must adhere to. For all tasks, novice participants used the lowest speed setting, while expert users were instructed to use their normal indoor speed settings so that performance was as natural as possible. Participants were never given performance-related feedback in between trials. Each trial was marked a pass or fail for the performance and safety components. The criteria performance criteria for safely conducted trials were taken according to the guidelines set in the user's manual of the WST 4.1 manual [17]. The results of each trial were recorded on a protocol sheet. The *Turns 90° While Moving *(forward and backward) and *Turns 180° in Place *tasks, as well as the *Maneuvers Sideways *task were conducted in both right and left directions. Each of these tasks and conditions (e.g., left/right, forward/backward) was repeated 3 times.

### Measurement of joystick control

**Figure 2:**
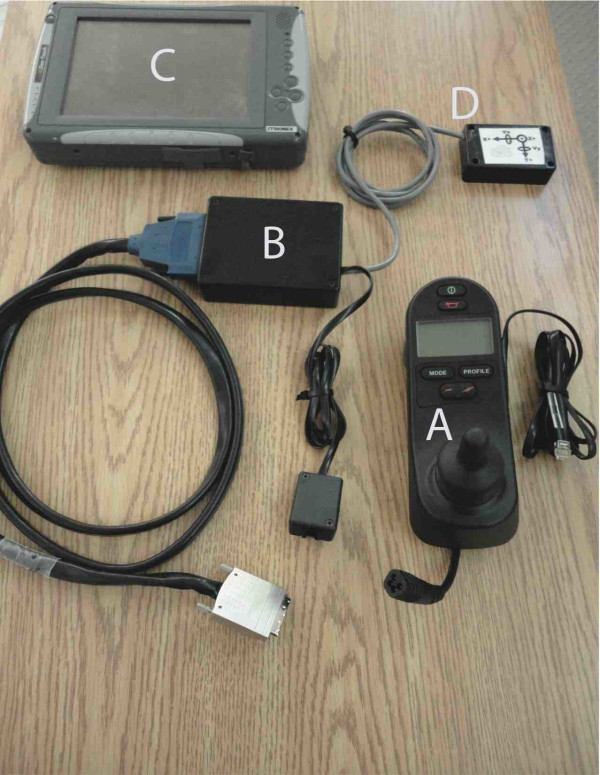
**Apparatus used for recording joystick control**. A: Modified joystick used to record biaxial (X-Y) movements, replaced the PW's original joystick of the same model. B: The central module receives biaxial joystick signals. C: Biaxial information is then sent to a tablet PC sampling at 200 Hz for data viewing and acquisition. D: Triaxial accelerometer and biaxial gyroscope (data not presented).

Before participants began the driving tasks, a lab-produced joystick controller (Figure 2A) was modified so that it could be interfaced with a data acquisition card (National Instruments 12-bit DAQCard-6024E) connected to a Tablet PC (Itronix, Duo-Touch) that was installed on the PW used for the testing (Figure 2C). The mechanical template of the joystick was circular so that movement in all directions was equidistant from the resting centre position. Joystick excursion about the centre (resting position) was measured. The joystick sent signals of joystick position in × and y components to the data acquisition board. Also attached to a central module (Figure 2B) was a tri-axial accelerometer (Figure 2D) fixed to a bar at the back of the wheelchair. For the expert group, the joystick was the same model as the hand-controlled joystick normally used by each participant. Any specialized handle (e.g., ball) needed by the participant was transferred to the joystick used for the experiment. The tablet PC was mounted at the rear of the wheelchair and another tablet PC was remotely synchronized so that the evaluator could remotely control which segments of data to record. Signals (X: left/right and Y: forward/backward) from the joystick were sampled at 200 Hz and recorded on the tablet PC using custom-made software.

### Data reduction and statistical analysis

**Figure 3:**
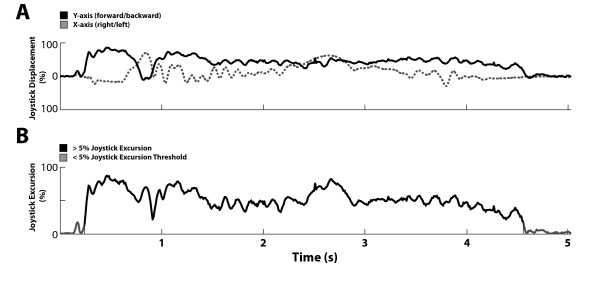
**Uniaxial and Biaxial interpretations of joystick displacement and combined excursion for a typical trial**. A: Uniaxial × (left/right) and Y (forward/backward) components of joystick displacement from the central resting position plotted over time during a single trial from the *Turns 90° While Moving Forward*. B: Biaxial (x and y components combined) representation for joystick excursions for the same trial.

The data were analyzed offline using custom routines developed in Matlab (The Mathworks, USA). The × (left/right) and Y (forward/backward) components of the joystick signals were first converted to polar coordinates to yield joystick excursion, or its absolute displacement from the central resting position (Figure 3A), and joystick direction. The number of joystick movements was defined as the number of times during a trial where the joystick excursion exceeded the threshold of 5% maximum displacement (see grey traces in Figure 3B) from the joystick's center position. Joystick excursion can be calculated by (x^2 ^+ y^2^)^1/2 ^where × is the horizontal (left/right) motion of the joystick and y is the vertical (forward/backward) component. Joystick orientation was calculated by tan^-1 ^(y/x). From this data, the total number of joystick movements needed to complete a trial could be computed. The total time required to execute each trial was defined as the movement time from the first to the last joystick excursion. For joystick direction, the raw data in each trial was first segmented into computationally convenient 100 ms time bins, which is half of the time of the minimal duration of a joystick movement (200 ms). The mean direction was calculated within each of these time bins. We then computed the intra-trial mean and variability of joystick direction based on these data. Inter-trial means and standard deviations were computed for trial duration, number of joystick movements and variability of joystick direction, for each subject and task. An independent t-test was then used to determine if there were significant differences (p < 0.05) in trial duration, number of joystick movements and variability of joystick direction between the novice and the expert groups for each task. Data collected from the accelerometer corresponding to the wheelchair's forward and backward movements were smoothed using a low-pass filter with a 5 Hz threshold (Butterworth, 5th order). Velocity could then be integrated from this data to compute the maximal forward and backward velocity of each participant. This was computed by taking the average peak velocity over all trials performed in the *Turns 90° While Moving Forward *and *Rolls Backward 5 m *tasks.

## Results

**Figure 4:**
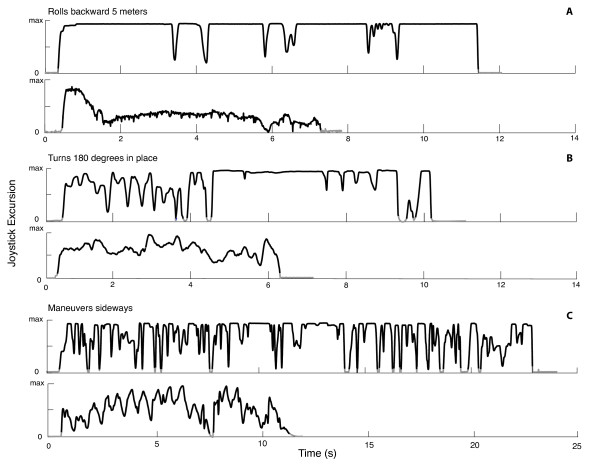
**Temporal schematic of joystick excursions for novice and experts users during individual PW tasks**. Biaxial joystick excursions for typical novice (top) and expert (bottom) users during A: *Rolls Backward 5 m *B: *Turns 180° in Place *and C: *Maneuvers Sideways *tasks. The black traces indicate when joystick excursion exceeded a resting threshold of 5%.

All subjects were able to complete all tasks successfully according to the WST (v.4.1) guidelines [17]. Individual trial data for typical novice and expert participants for *Rolls Backward 5 m*, *Turns 180° in Place*, and *Maneuvers Sideways *tasks are illustrated in Figure 4A-C respectively. With joystick excursions visually plotted in this manner, clear distinctions can be made between a typical novice and expert user with respect to joystick control (number of movements) and task completion time.

### Right and Left Trial Comparisons

A paired t-test was conducted to compare tasks with left and right variations. These tasks included the *Turns 90° While Moving (forward or backward), Turns 180° in place*, and *Maneuvers Sideways *tasks. In all of these tasks, no significant differences were found between right and left sided trials for any of the measured outcomes mentioned above (p > .05). This enabled us to combine right and left sided trials with respect to measuring the number of joystick movements, task completion time and directional variability.

### Joystick Movements

**Figure 5:**
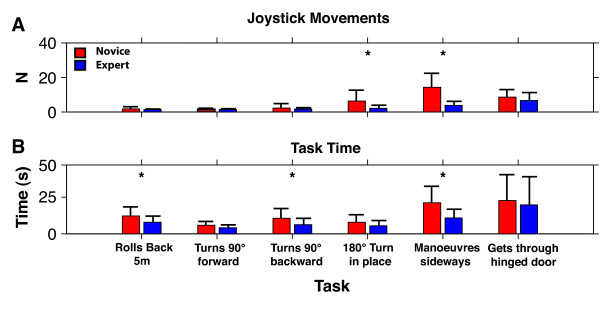
**Mean trial joystick movements and time durations**. Means (± SD) of novice (red) and expert (blue) performances across all six tasks for A: the number of joystick movements to complete the task and B: The amount of time required to complete the task.

Figure 5A illustrates the mean number of joystick movements across all six tasks for the novice (red) and expert (blue) groups. In these trials, both novice and expert users required similar amounts of joystick movement for the *Rolls Backward 5 m *and the *Turns 90° While Moving Forward *or *Backward *tasks *(p > .05)*. Mean values are also shown in Table 2. When comparing *Turns 180° in Place *and the *Maneuvers Sideways *tasks, we observed significant differences in joystick control strategies and performance between groups. The expert group required fewer joystick movements for the *Turns 180° in Place *and *Maneuvers Sideways *tasks (p < .001). The mean number of movements was approximately four times greater in the novice group relative to the experts in both tasks (refer to Table 2). For the *Gets Through Hinged Door *task, no statistical difference was found between groups in terms of number of joystick movements required (p > .05).

**Table 2:** Mean values of outcome measures for novice and expert groups

	Novice Mean (±SD)	Expert Mean (±SD)	P value	Effect-Size
*Number of joystick excursions*				
Rolls backward 5 meters	1.79 (1.80)	1.36 (.31)	n.s	0.33
Turns 90° (forward)	1.50 (.67)	1.38 (.42)	n.s	0.21
Turns 90° (backward)	2.25 (1.56)	1.66 (.78)	n.s	0.48
Turns 180° in place	9.67 (6.30)	2.07 (1.76)	p < .001	1.64
Manoeuvres sideways	14.26 (8.10)	3.82 (2.30)	p < .001	1.75
Gets through hinged door	8.58 (4.46)	6.61 (4.57)	n.s	0.44
*Task time (sec)*				
Rolls backward 5 meters	12.93 (6.59)	8.41 (4.46)	p < .05	0.80
Turns 90° (forward)	6.26 (2.73)	4.40 (2.14)	n.s	0.76
Turns 90° (backward)	11.29 (7.07)	6.63 (4.59)	p < .01	0.78
Turns 180° in place	8.36 (5.45)	5.80 (3.81)	n.s	0.54
Manoeuvres sideways	22.60 (11.94)	11.50 (6.38)	p < .001	1.16
Gets through hinged door	24.09 (18.80)	21.02 (20.42)	n.s	0.16
*Directional Variability*				
Rolls backward 5 Meters	14.56 (10.56)	17.68 (6.49)	n.s	-0.36
Turns 90° (forward)	30.59 (7.14)	31.48 (13.36)	n.s	-0.08
Turns 90° (backward)	30.83 (20.09)	24.56 (13.95)	n.s	0.36
Turns 180° in Place	57.09 (9.24)	52.29 (15.09)	n.s	0.38
Manoeuvres Sideways	71.74 (1.63)	76.51 (2.67) n.s	-2.1	
Gets Through Hinged Door	65.49 (10.12)	66.87 (8.37)	n.s	-0.14

Grand means for each sub-task for novice and expert groups. p values were calculated using an independent t-test between novice and expert groups for each sub-task. Cohen's D values were used for reporting effect size

### Task Completion Time

Figure 5B illustrates the mean trial completion times for novice and expert groups across all tasks. Similar mean time performances were observed for the *turns 90° While Moving Forward *task (p > .05). However, for the time required to complete the *Rolls Backward 5 m*, and *Turns 90° While Moving Backward *tasks, a statistical difference (p < .05) suggests that the expert group generally completed these reverse tasks more quickly than their novice counterparts.

The novice group generally took the same amount of time to complete the *Turns 180° in Place *task relative to the expert group (p > .05) (Figure 5B). On the other hand, the expert group performed significantly better than the novice group (p < .001) for the *Maneuvers Sideways *task, on average completing this task in 11.5 seconds - approximately half the time taken by novice participants. For the *Gets Through Hinged Door *task, both groups took the same amount of time to complete the task (p > .05).

### Mean Directional Variability

**Figure 6:**
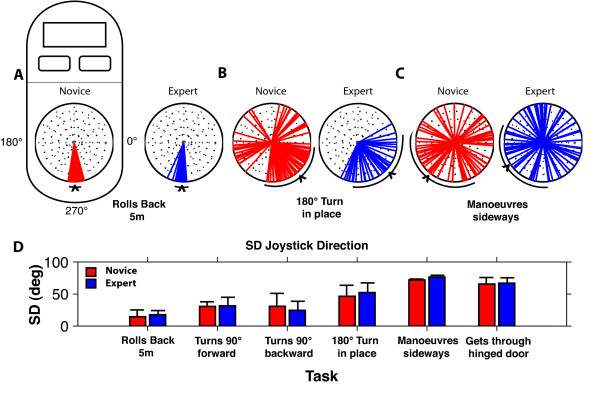
**Mean joystick movements and variability**. Mean (± SD) joystick direction across trials for a typical novice (left) and expert (right) for A: *Rolls Backwards 5 m *B: *Turns 180° in Place *and C: *Maneuvers Sideways *tasks. Each vector represents the direction during a 100 ms data bin. D: Mean (± SD) joystick directional variability for novice (red) and expert (blue) participants for each task. The arc under each joystick (A, B and C) represents a single standard deviation of the vector scatter plotted on the joystick, while the v marks the value of the mean.

Figure 6A-C illustrates the distribution of angular joystick direction for a novice and an expert participant in the *Rolls Backward 5 m*, *Turns 180° in Place*, and *Maneuvers Sideways *tasks. Both the novice and expert subjects showed similar joystick trajectories for the *Rolls Backward 5 m *task (Figure 6A). For tasks requiring more frequent changes in direction, such as the *Turns 180° in Place *and *Maneuvers Sideways *tasks (Figure 6B-C), the distribution of joystick direction was broader with a larger variability.

Figure 6D illustrates the mean directional variability of the novice and expert groups across the six tasks aforementioned. Directional variability for all of these tasks proved to be rather comparable between the groups, showing no statistical significance for any of the tasks (p > .05; see Table 2).

Trial failure rates were recorded for trials that were recorded using the data logger. Overall, novice users failed 20.0% (±12.1%) of their recorded task trials compared to only 10.8% (±5.7%) of failed recorded trials conducted by experts (p = .05).

Both forward and backward maximal velocities (meters per second) were computed using the *Turns 90° While Moving Forward *for the forward maximal velocity and the *Rolls Back 5 m *tasks respectively. This analysis was done to determine whether varying PW speeds made one group travel faster than the other. For the maximal backward velocity, the mean velocity for the expert group was 0.88 (±0.26), while the novice group average was 0.79 (±0.09). For the maximal backward velocity, the expert group average was -0.44 (±0.13), and the novice group was -0.43 (±0.11). Independent samples t-tests confirmed that no significant differences were found between the groups, for either maximal forward or backward velocity (p > 0.05).

Further analysis was conducted to estimate whether learning effects were present in expert or novice subjects as a result of repeating tasks. By excluding the first trial of every task for each subject, we found no evidence of learning effects compared to when the first trial was included. Moreover, when comparing expert and novice performances with the first trial removed, very similar results were yeilded apart from the time to complete the *Turns 90° Backward *task (p > .05).

## Discussion

The goal of this study was to estimate the extent to which data logging could be used to discriminate PW driving skills in experienced users relative to novice users when completing a series of standardized tasks in a motorized wheelchair. We recruited novice users who reported never using a PW before and pitted their driving skills to more experienced users. These first time users were recruited to represent a new PW user's driving potential. To this end, not only could we estimate the difference in skill level of joystick operation between the two groups, but also outline some of the common challenges that the novice user might face when learning to operate a PW. Using joystick data in tandem with an observational approach such as the one used in this study can contribute to optimizing training strategies for those who require the use of a PW but are new to operating one.

For relatively simple PW tasks, such as *Rolls backward 5 m *and *90° Turns While Moving Forward*, the extent to which the novice group, using a PW for the first time, was able to perform such tasks effectively is seemingly comparable to expert users. Specifically, both groups seemed to require similar amounts of joystick movements, while also completing these tasks in a fairly analogous time frame. Perhaps performing these tasks in optimal conditions (i.e. flat surface, no pedestrians/traffic) may have contributed to the similar joystick control strategies and performance in both groups. In fact, it was only during more challenging and spatially confined tasks, such as the *Turns 180° in Place *and *Maneuvers Sideways*, that expert users tended to exhibit greater dexterity relative to their novice counterparts. This is evident in the expert group's reduced joystick movements and time required to complete such tasks. In some instances, these differences were quite marked as joystick excursions for experts were generally reduced to about half with respect to their novice counterparts. The *Gets Through Hinged Door in Both Directions *task could be also considered a relatively challenging task. Surprisingly, the novice group seemed to complete this task almost as well as the expert group (see table 2). It is possible that this task affected both groups in a different way. For example, all expert users had disabilities affecting the lower extremities and most had disabilities affecting trunk and/or upper extremity control. Thus, they may have been skilled at controlling the PW, but were faced with adaptation challenges when interacting with the environment (i.e. maintaining trunk stability while reaching for the doorknob). Conversely, the novice participants simply had to cope with a novel and relatively involved task, but could compensate with a longer reach by bending the trunk forward or sideways, as required. It is possible that the respective difficulties encountered by both groups in this task lead to comparable joystick control strategies and performance.

Measuring joystick directional variability did not seem to differ between groups, regardless of task difficulty. Nonetheless, these directional variability results suggest that a modification may be required to optimize its effectiveness as a measurement tool. Appropriate modifications to joystick variability measures could perhaps also yield more valid and interesting findings.

To avoid comparing the different dynamics of rear-wheeled and mid-wheeled PWs, rear-wheeled wheelchairs were used in the study since the majority of PWs used in Québec are rear-traction. This may pose as a limitation to our findings since we can not generalize them beyond the rear-traction PW. Since rear-wheeled PWs tend to operate less agilely in tight quarters compared to their mid-wheel analogue, perhaps the rear-wheeled performance observed in the study transfers well to the mid-wheelchair. All of the novice participants used the lowest speed, yet we chose not to control for expert PW speeds because we wanted the expert group to perform driving tasks as they would in their daily lives. Perhaps this poses as a limitation in the methodology. Despite the varying speeds used, no significant differences were found between expert and novice groups with respect to forward and backward velocity. Consequently, we believe that the speed setting differences do not account for the results reported in the time to complete tasks and the number joystick movement measures. In a similar vein, we wanted the expert participants to perform tasks with their normal PW programmed settings. It is possible that some experts used a smaller joystick excursion to attain the same speed. We do not feel that differences in joystick sensitivity could have affected our results, namely the computation of the number of joystick movements, as this was set at a low joystick excursion threshold (5%).

In drawing conclusions from this study, it must be considered that this was a pilot study with a small sample size and that there were no a priori data to estimate effect sizes. As a result, the effect size of the statistical analyses performed varied from .08 to 2.1, which could explain the lack of significant differences for the simpler tasks, such as the 90° turns. Furthermore, it is possible that the metrics used as outcome measures (i.e. number of joystick movements, direction of movement and total time required to execute each trial) may not have the necessary sensibility to discriminate between novice and expert users for the simpler tasks, due to their short duration [27]. It is possible that more sensitive metrics could be devised, based on other metrics and on data from different types of sensors (e.g. accelerometers). It remains to be seen whether such measures can be clinically relevant. From a clinical standpoint, it might be sufficient to know if a participant is able to perform simple driving tasks or not, for the purpose of deciding whether the person can then be trained to safely drive a PW. Quantitative information about performance may be useful for the more complex PW driving tasks in order to provide better guidelines for training.

In this experiment, the measurement of joystick control was provided by a data-logging platform, which also includes other sensors such as accelerometers, gyroscopes, a wheel encoder, seat pressure sensors and GPS [8,23]. The use of a data logging platform in combination with such sensors can complement observation-based methods of assessing PW driving performance. Offering insights on joystick control strategies could expose users to better and safer driving techniques early on in the learning process. Such outcome measures could also be used as feedback to the new PW user and serve as benchmarks for specific task execution, while also helping to prioritize training components. Future studies will employ more participants and focus on assessing the efficiency of training protocols for PW users, combining both observational and data logging methods. Building on the results of this study, future work can evaluate the effect of training new PW users on a training program by providing ongoing performance feedback with respect to wheelchair tasks. Providing users with such feedback could expose them to better and safer driving techniques early on in the learning process.

## Conclusion

In general, tasks drawn from the WSP that are typically associated to more difficult skills tend to show differences in joystick control strategies and performance between expert and novice groups. In particular, the expert group displayed reduced joystick excursions and task completion times compared to their novice counterparts. Lastly, data from movement-sensing joysticks used on PWs during selected driving tasks could provide an effective technique for quantifying key aspects of PW driving skills. Thus, the combination of objective measurement of PW control using joystick data in tandem with observational strategies may be an effective tool for the clinical assessment and training of PW driving skills.

## List Of Abbreviations

PW: Powered wheelchair; WST-P: Wheelchair Skills Test, Powered Wheelchair Version.

## Competing interests

The authors declare that they have no competing interests.

## Authors' contributions

GS contributed to the data collection. Both GS and PA contributed to participant recruitment, data analysis, interpretation of results, and manuscript production. FR and PB participated in the study design and reviewed the manuscript. DD contributed to participant recruitment. All authors read and approved the final manuscript.
